# Contemporary evidence of long-term follow-up of the Epic^TM^ prosthesis after mitral valve replacement

**DOI:** 10.34172/jcvtr.025.33387

**Published:** 2025-09-28

**Authors:** Beatriz Acuña Pais, Daniel Otero Lozano, Consuelo María Sisinni Ganly, Carolina Mayor Deniz, Rocío Casais Pampín, Juan José Legarra Calderón

**Affiliations:** ^1^Cardiovascular Surgery Service, Álvaro Cunqueiro Hospital, Vigo, Spain; ^2^Thoracic Surgery Service, Álvaro Cunqueiro Hospital, Vigo, Spain

**Keywords:** Mitral valve, Replacement, Outcomes, Biocor, Epic

## Abstract

**Introduction:**

To verify the long-term durability, freedom from reoperation and mortality of Biocor and Epic bioprosthesis in mitral position.

**Methods:**

The use of biological prostheses in mitral valve replacement surgery is widespread. Advances in transcatheter techniques have increased the utilisation of these biological substitutes, rendering the study of their durability once again pertinent. Biocor and Epic are two stented tissue valves with porcine leaflets indicated for patients requiring replacement of a diseased mitral or aortic heart valve. Long-term follow-up data were collected and analysed by age group and by type of surgery. Between 2000 and 2010, 244 patients underwent mitral or double valve replacement with a Biocor or Epic bioprosthesis at our institution. The median follow-up was 9 years (IQR: 0.01- 17.9). Complete follow-up was achieved in 98.4%.

**Results:**

The survival rates at 1, 5 and 10-years were 90, 73.7 and 43 %, respectively. Freedom from prothesis reintervention from any cause at 1, 5 and 10 years was 98.5, 92.8 and 90%. Freedom from structural valve deterioration by age group at 1, 5 and 10 years was as follows: age<60: 100, 84.6 and 47.6; age 60-69: 100, 94.1 and 94.1; and age≥70: 99.4, 98.7 and 96.5% respectively.

**Conclusion:**

Our 10-year freedom from structural valve deterioration of 93.1% matches, and may even surpass those previously reported. The Epic porcine xenograft in mitral position has demonstrated to have excellent durability and long-term outcomes; representing an excellent option for patients in need for mitral valve replacement.

## Introduction

 The use of biological prostheses in mitral replacement surgery is widespread. Advances in transcatheter techniques have increased the implantation of these biological substitutes, making the study of their durability once again relevant.

 Based on the patient’s individual characteristics, we will choose between mechanical or biological valves. The former require lifelong anticoagulant therapy with a higher risk of bleeding complications, whilst the latter carry the potential risk of structural valve deterioration due to tissue failure; hence, these prostheses are an interesting choice in individuals older than 65.^1^

 Biocor^R^ (St Jude Medical, St Paul, Minn) and Epic^TM^ (Abbott cardiovascular Inc., St Paul, Minn), are two stented tissue valves with porcine leaflets indicated for patients requiring replacement of a diseased mitral or aortic heart valve. The Epic valve is identical to the Biocor, but is treated with the Linx anti-calcification process and is therefore expected to perform better.

 The good long-term performance of the Biocor prosthesis in mitral and aortic valve positions has already been verified in other studies.^2-4^ Also the durability of the Epic valve.^5-9^

 To verify the long-term durability of both prostheses, we retrospectively studied the results of patients who underwent mitral valve replacement alone or in combination with aortic valve replacement in our department. Our study focuses on evaluating freedom from reoperation and mortality related to valvular degeneration. We analysed results based on the two groups of surgical patients, MVR and DVR, and differences between three age groups (< 60, 60–69, ≥ 70 years).

 To evaluate long-term results after mitral or double valve replacement with a second- and third-generation porcine bioprosthesis, Biocor and Epic valves.

## Materials and Methods

###  Study population

 Between January 2000 and December 2010, 244 consecutive patients underwent mitral valve replacement (MVR) or double (mitral and aortic) valve replacement (DVR) with a Biocor or Epic bioprosthesis at our institution. The Biocor prosthesis was implanted between 2000 and 2007, and the Epic prosthesis from 2007 onwards.

 Of these patients, 160 underwent MVR alone and 84 underwent DVR. All patients had indications for elective or emergency procedures, and all indications for MVR were included.

###  Follow-up

 Demographic, clinical, echocardiographic and follow-up data were retrospectively obtained from the medical records of our hospital. This retrospective analysis received institutional-review-board-permission number 2021/521.

###  Definitions of events 

 This study was conducted in accordance with the Guidelines for Reporting Morbidity and Mortality after Cardiac Valve Interventions.^10^ Akins’ recommendations were not strictly followed for the definition of structural valve deterioration (SVD) due to the lack of echocardiographic data.SVD was defined as any dysfunction or deterioration involving the prosthesis (exclusive of infection or thrombosis) as determined by reoperation/ indication for reoperation or death due to SVD.

 Reoperations met the study endpoints when motivated by any dysfunction of the mitral bioprosthesis. Mitral valve-related mortality was defined if occurring dysfunction or infection of the bioprosthesis, or reoperation on the mitral valve. We distinguished between surgical reoperation and transcatheter mitral valve-in-valve to treat SVD.

 Operative mortality and cardiac reoperation were defined as occurring within the 30^th^ postoperative day, or later if during the same hospitalisation. In late follow-up analysis, the population was stratified into two subgroups by index surgery: MVR or DVR.

###  Endpoints

 Short and long-term outcomes of MVR with Biocor and Epic prosthesis. Early and late follow-up results of related and unrelated valve events, survival (overall and stratified according to age subgroups) among MVR recipients using the Epic bioprosthesis.

###  Statistical Analysis 

 Qualitative variables were compared by the chi-square test or Fisher exact test; quantitative variables were compared by the Student t-test or Mann-Whitney test.

 Quantitative data are presented as mean SD (if normally distributed) or as median [IQR] (if non-normally distributed). Categorical variables are reported as percentages. Quantitative variables were compared by the Student t-test or Mann-Whitney test. Qualitative variables were compared by the Pearson’s Chi-square test or Fisher’s exact test. The Kolmogorov-Smirnov test assessed normal distribution of continuous variables.

 Kaplan-Meier analyses were performed for survival or freedom from the event rates, and groups compared using the log-rank static.

 Survival was analysed using the Kaplan-Meier survival estimate, with log-rank for comparisons. The alpha value was 0.05.

 The study adheres to the STrengthening the Reporting of OBservational studies in Epidemiology (STROBE) initiative.

 Statistical analysis was performed using the SPSS statistical program, version 19.0 (IBM, 2010).

## Results

###  Patients 

 For the period from 2000 to 2010, we retrospectively analysed all patients who underwent primary MVR or DVR with Biocor or Epic prosthesis. The median age was 75 (IQR: 23-86) years, and 47.5% were male. The preoperative characteristics are detailed in Table 1.

**Table 1:** Preoperative characteristics

	**Total**	**Mitral valve replacement**	**Double valve replacement**	**Significant**
n	244	160 (65.6)	84 (34.4)	-
Age, y				
< 60 y, (%)	14 (5.7)	9 (5.6)	5 (5.6)	-
60-69 y, (%)	26 (10.7)	20 (12.5)	6 (7.1)	-
≥ 70 y, (%)	204 (83.6)	131 (81.9)	73 (86.9)	0.436
Male sex, %	116 (47.5)	82 (51.3)	34 (40.5)	0.109
Arterial hypertension, %	140 (57.4)	90 (56.3)	50 (59.5)	0.623
Non-insulin dependent diabetes mellitus, %	43 (17.6)	28 (17.5)	15 (17.9)	0.945
Insulin dependent diabetes mellitus, %	8 (3.3)	6 (3.8)	2 (2.4)	0.718
Chronic obstructive pultmonary disease, %	37 (15.2)	25 (15.6)	12 (14.3)	0.782
Renal insufficiency, %	24 (9.8)	15 (9.4)	9 (10.7)	0.739
Peripheral vascular disease, %	13 (5.3)	11 (6.9)	2 (2.4)	0.229
Cerebrovascular accident, %	20 (8.2)	12 (7.5)	8 (9.5)	0.584
Atrial fibrillation, %	131 (53.7)	85 (53.1)	46 (54.8)	0.808
Previous cardiac surgery, %	37 (15.2)	21 (13.1)	16 (19)	0.220
Previous valvular surgery, %	30 (12.3)	19 (11.9)	11 (13.1)	0.200
Active infective endocarditis, %	26 (10.7)	17 (10.6)	9 (10.7)	0.983
Previous valvular surgery and active infective endocarditis, %	8 (3.3)	5 (3.1)	3 (3.6)	1.000
Timing				
Elective, %	166 (68)	107 (66.9)	59 (70.2)	0.592
Urgent, %	70 (28.7)	45 (28.1)	25 (29.8)	0.788
Emergency, %	8 (3.3)	8 (5)	-	-

###  Procedural details

 Four patients underwent aortic valve replacement (AVR) with a prosthesis different from Biocor or Epic, in such cases we included their data in the group of single MVR with concomitant procedure. One patient in the MVR group also received an aneurysmectomy procedure.

 Procedural details are shown in Table 2.

**Table 2:** Procedural destilas

	**Total**	**Mitral valve replacement**	**Double valve replacement**	**Significant**
Concomitant procedure, n (%)	112 (45.9)	74 (46.3)	38 (45.2)	0.880
Tricuspid valve repair, n (%)	63 (25.8)	44 (27.5)	19 (22.6)	0.408
CABG, n (%)	44 (18)	28 (17.5)	16 (19)	0.765
Ascending aorta replacement, n (%)	7 (2.9)	2 (1.3)	5 (6)	0.049
Atrial ablation, n (%)	34 (13.9)	22 (13.8)	12 (14.3)	0.909
Left atrial appendage closure, n (%)	48 (19.7)	34 (21.3)	14 (16.7)	0.392
Cardiopulmonary bypass time (CPB)(min)	195.98 ± 81.8	176 ± 86.6	234 ± 55	< 0.001
Aortic cross-clamp time (min)	162.5 ± 65	141.9 ± 64.2	200.5 ± 46.9	< 0.001
MVR with Biocor, n (%)	119 (48.8)	77 (48.1)	42 (50)	0.781
MVR with Epic, n (%)	125 (51.2)	83 (51.9)	42 (50)	0.781
AVR with Biocor, n (%)	-	-	40 (47.6)	-
AVR with Epic, n (%)	-	-	44 (52.4)	-
Mitral valve size	29.6 ± 1.7	29.9 ± 1.7	29 ± 1.7	< 0.001
Aortic valve size	-	-	22.3 ± 1.4	-

###  Primary Outcomes

####  Long-term Survival

**Figure 1:**
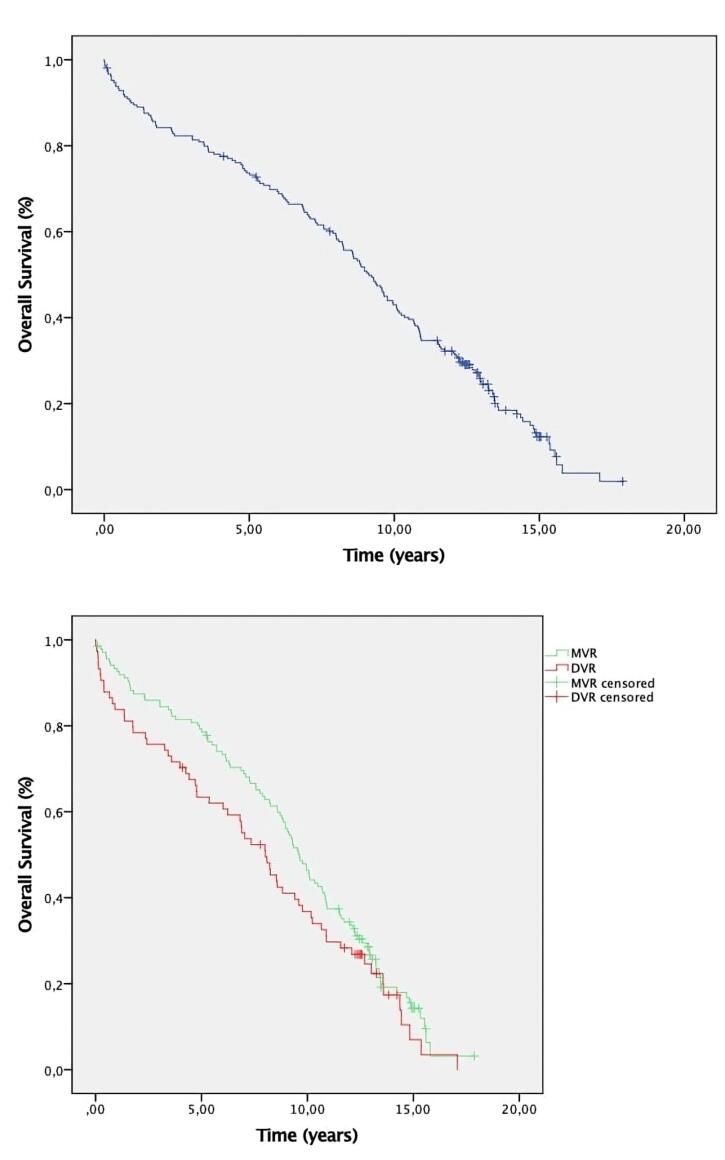
A. Kaplan- Meier survival curve for the total of patients at follow-up B. Kaplan- Meier survival curve for mitral and double valve replacement patients at follow-up

 Median follow-up was 9 years (IQR: 0.01–17.9). Four patients were lost to follow-up, resulting in a complete follow-up rate of 98.4%. A total of 2,684 patient-years were available for analysis. The survival rate at 1, 5, and 10-year follow-up was 93.3%, 79.3%, and 46.4% for MVR; 83.8%, 63.4%, and 36.8% for DVR; and 90%, 73.7%, and 43% for the total cohort. There was no meaningful difference in survival at follow-up among groups. Figure 1 A shows Kaplan- Meier survival curve for the total of patients at follow-up. Figure 1 B shows Kaplan- Meier survival curve for MVR and DVR patients at follow-up.

 Causes of mortality during follow-up were: cardiac not prosthesis-related 21.6% (37), other causes 56.1% (96), unknown 14.6% (25), endocarditis 6.4% (11), and prosthesis-related 1.2% (2). Other causes of death include respiratory, oncological, neurological and mesenteric ischemia.

####  Freedom from Prosthesis Reintervention

 Reoperations over time were due to infective endocarditis in 10 patients (4.8%) and degenerative process in 9 (4.3%) (1 transcatheter and 8 open surgical procedures). Four patients (1.9%) had indication for reoperation (2 endocarditis and 2 degeneration) but were not admitted to surgery due to comorbidities.

 Early reoperation ( < 1 year after initial procedure) was performed in 2 patients (1%): one due to endocarditis and one due to SVD.

 Freedom from prosthesis reintervention from any cause at 1, 5, and 10 years was 98.5%, 92.8%, and 90%, respectively.

 Freedom from prosthesis reintervention from any cause by age group at 1, 5 and 10 years were as follows: age < 60: 100, 76.9 and 51.3%; age 60-69: 100, 94.1 and 94.1; and age > 70: 98.2, 94.1 and 93.3 respectively. It was significantly higher in patients aged < 60, as shown in Table 3.

**Table 3:** Freedom from prosthesis reintervention and structural valve deterioration by age group

**Characteristic**	**Age<60**	**Age 60 to 69**	**Age≥70**	***P***
Freedom from prosthesis reintervention	38.5%	90.9%	93%	< 0.001
Freedom from SVD	53.8%	86.4%	94.2%	< 0.001

SVD = Structural valve deterioration

**Figure 2:**
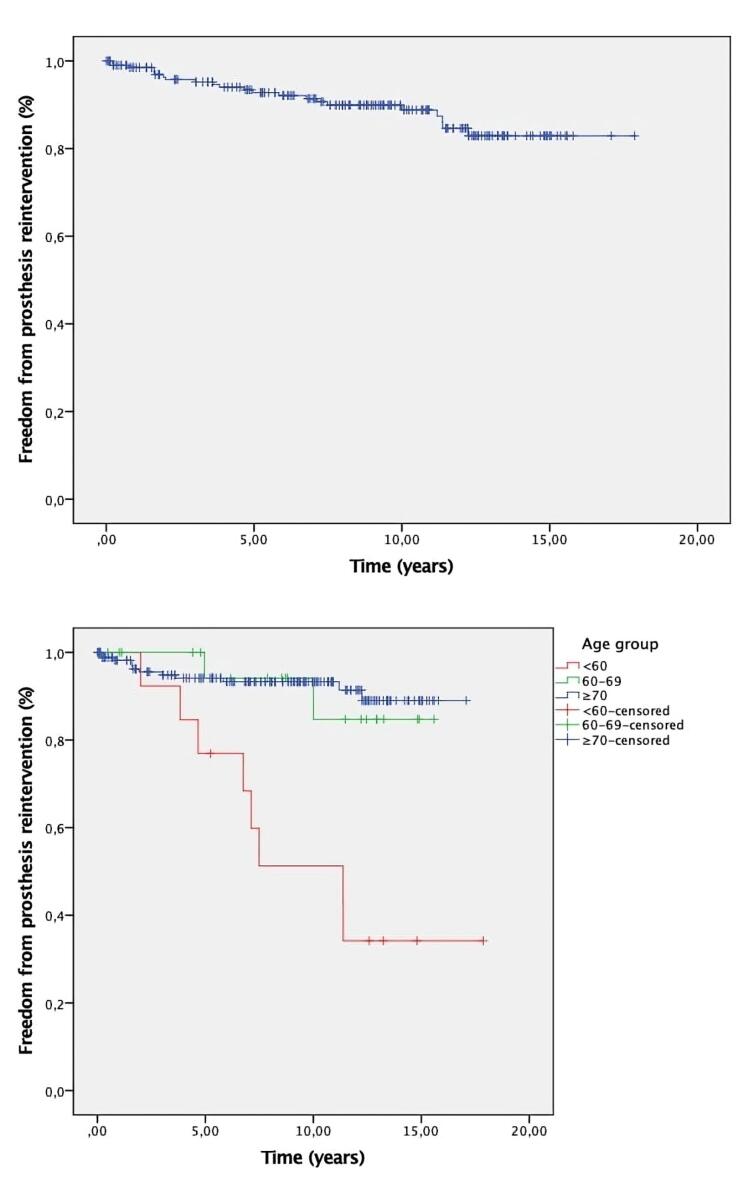
A. Kaplan-Meier curve for freedom from prosthesis reintervention B. Kaplan-Meier curve for freedom from prosthesis reintervention by age group

Figure 2 A shows the Kaplan-Meier curve for freedom from prosthesis reintervention. Figure 2 B shows the Kaplan-Meier curve for freedom from prosthesis reintervention by age group.

####  Freedom from Structural Valve Deterioration (SVD)

 Median echocardiographic follow-up was 6.8 years (IQR: 0.01- 17.7).

 According to our study definition, SVD (any dysfunction or deterioration involving the prosthesis exclusive of infection or thrombosis, as determined by reoperation/indication for reoperation or death) was found in 14 patients (6.7%). Three patients (1.4%) were on the waiting list for reoperation at the time of the study.

 Freedom from SVD at 1, 5, and 10 years was 99.5%, 97.1%, and 93.1%, respectively.

 By age group: < 60 years: 100%, 84.6%, and 47.6%; 60–69 years: 100%, 94.1%, and 94.1%; ≥ 70 years: 99.4%, 98.7%, and 96.5%.

 Structural valve deterioration was significantly higher in patients < 60, as shown in table 3.

**Figure 3:**
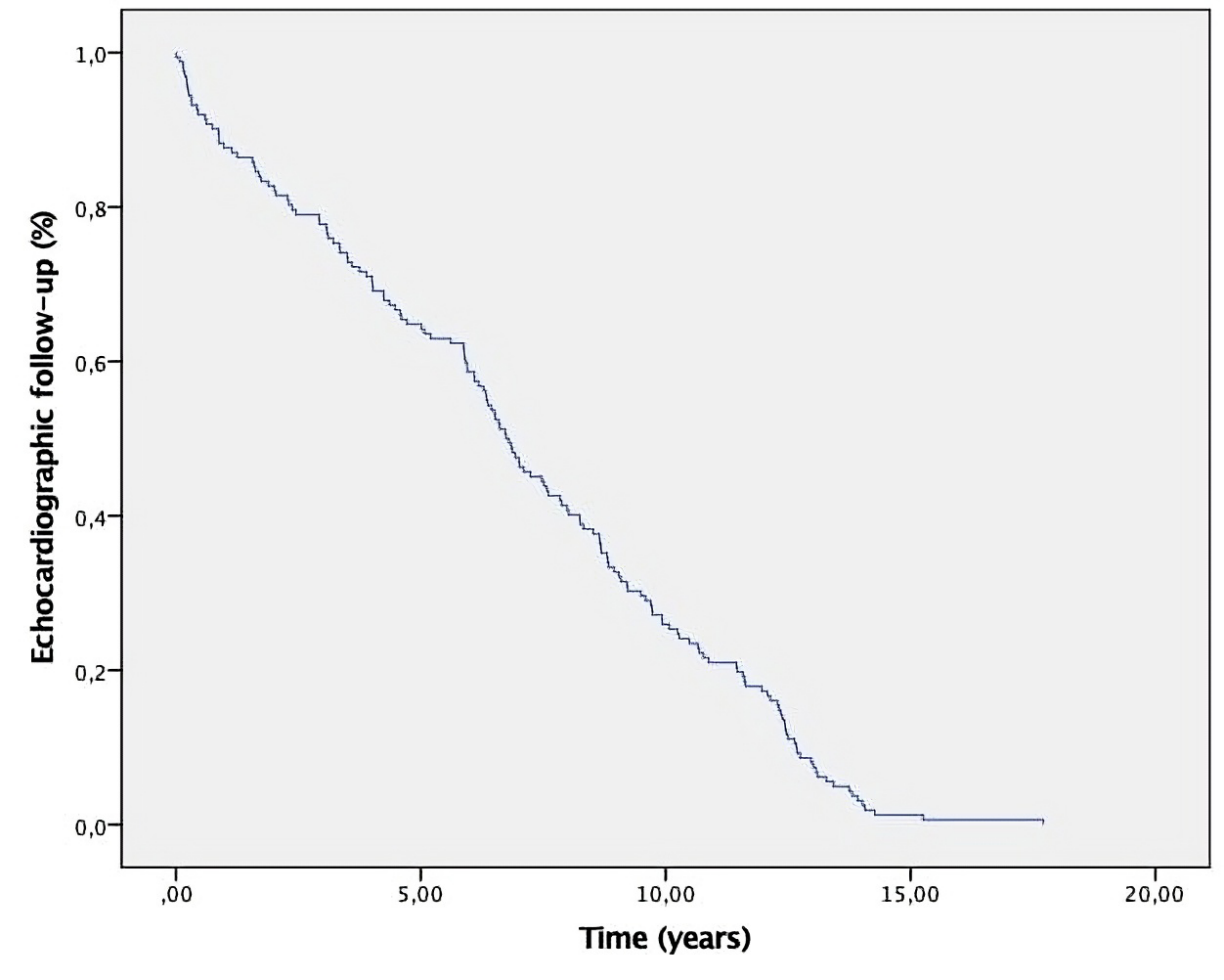
Kaplan-Meier curve for echocardiographic follow-up

**Figure 4:**
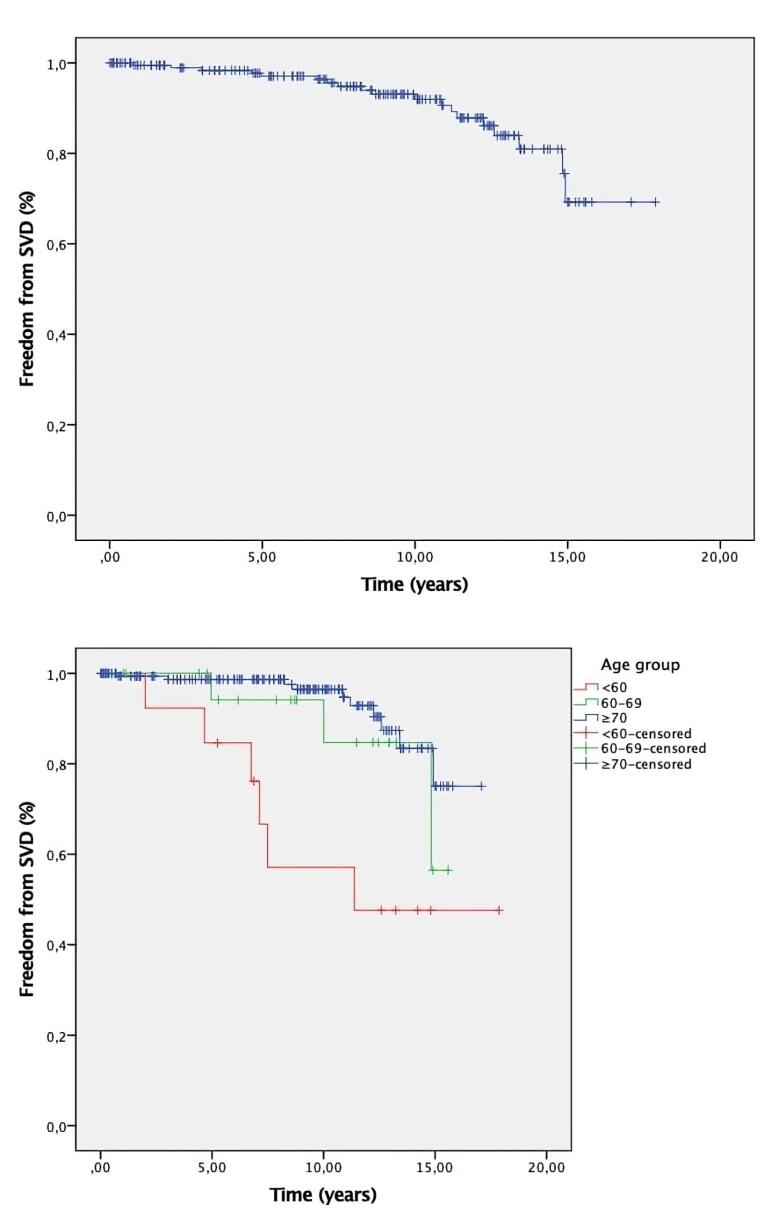
A. Kaplan-Meier curve for structural valve deterioration B. Kaplan-Meier curve for freedom from structural valve deterioration by age group

Figure 3 shows the Kaplan-Meier curve for echocardiographic follow-up. Figure 4 A shows the Kaplan-Meier curve for SVD. Figure 4 B shows the Kaplan-Meier curve for freedom from SVD by age group.

###  Secondary Outcomes

####  Early Postoperative Complications

 Postoperative respiratory failure requiring intubation over 24 hours occurred in 16.3% (MVR) and 19% (DVR) (*P* = 0.582). The rate of re-exploration for bleeding was 3.1% (MVR) and 10.7% (DVR) (*P* = 0.021). The rate of subxiphoid drainage for pericardial tamponade was 3.1% (MVR) and 3.6% (DVR) (*P* = 0.852). Cerebrovascular accidents occurred in 6.3% after MVR and 1.2% after DVR (*P* = 0.103). Acute kidney failure requiring temporary haemodialysis occurred in 11.9% (MVR) and 13.1% (DVR) (*P* = 0.783). Pacemakers were implanted in 6.3% and 14.3% of patients undergoing MVR and DVR, respectively (*P* = 0.037). The mean lengths of hospital stay were 17.4 ± 39.2 days (MVR) and 17.7 ± 17.8 days (DVR) (*P* = 0.956). Twenty-five (15.6%) patients undergoing MVR and twelve (14.3%) undergoing DVR died while in hospital postoperatively (*P* = 0.782). Eighteen patients died due to septic shock, 12 of whom had endocarditis. One patient undergoing MVR died after discharge within 30 days postoperatively. None of these deaths were valve-related.

####  Risk Factors for Mortality and SVD

 With regard to hospital mortality, the factors with p < 0.005 on univariate analysis were: previous cardiac surgery, urgent/emergent surgery, active infective endocarditis, aortic cross-clamp time > 180 min, CPB > 200 min, prolonged intubation, postoperative haemodialysis, and postoperative cerebrovascular accident. According to multivariate analysis including these factors, active infective endocarditis (HR 9.37; 95% CI: 2.065–39.550, *P* = 0.003) and prolonged intubation (HR 6.796; 95% CI: 2.533–18.233, *P* < 0.001) were independent predictors.

 Cox proportional hazards model demonstrated the following significant covariates for increased mortality at final follow-up: arterial hypertension (HR: 1.463 [95% CI: 1.030–2.079]; *P* = 0.034), diabetes mellitus (HR: 2.004 [95% CI: 1.318–3.047]; *P* = 0.001).

 Age < 60 years resulted in a protective factor for mortality at final follow-up (HR: 0.24 [95% CI: 0.081–0.708]; *P* = 0.01).

 Cox proportional hazards model demonstrated that age < 60 years resulted in significant increase of prosthesis degeneration at final follow-up (HR 5.360; 95% CI: 1.516–18.947, *P* = 0.009).

## Discussion

 Mitral valve replacement represents 40% of all mitral valve surgeries.^11^

 According to the latest ESC guidelines on valvular heart disease, a bioprosthesis should be considered in patients aged > 70 years and a mechanical prosthesis in those aged < 65 in the mitral position, leaving a gap regarding the optimal prosthesis for patients between 65 and 70 years.^1^

 The high anatomo-clinical complexity of our series is reflected by the characteristics of our patients: age > 70 (83.6%), DVR (34.4%), infective endocarditis (11%), previous cardiac surgery (15%), COPD (15%), previous cerebrovascular accident (10%), renal failure (10%) and DM (10.9%). We report a 15.1% mortality, similar to other studies with comparable populations.^3^

 Our study shares a robust follow-up with 2684 patient-years, and mean follow-up of 9 years, higher numbers than many other studies, providing great power to our results.

 Survival at 10 years is similar to other studies.^3,6^ Age was not related to long-term mortality, also reflected by Anselmi et al.^8^ Regarding long-term mortality, only in 1.2% of cases it was prosthesis-related; most of patients died due to other causes, as studied earlier.^3^

 Prosthesis degeneration does not necessarily mean reintervention. Many SVD cases only required follow-up. Additionally, a small number of patients with indication for reintervention are not accepted because of high comorbidities. In the case of other bioprothesis, freedom from SVD is between 45-84% at 10 years, compared to 75-95% at 8-10 years for Biocor, and up to 98% at 10 years for Epic.^3,6^ The Epic prosthesis has a promising haemodynamic performance and durability.^5-7^ In our case, freedom from SVD was 93% at 10 years. Lehman et al reported similar results, 93.8 % for single replacement, and 92.8% for DVR.^9^ In our SVD analysis by age group, we clearly observed a higher rate of SVD in patients < 60 years, as expected and already described by Akins et al^10^ We found a 11% of SVD in the group of patients between 60-69 years, with a freedom from SVD of 86.4% at 10 years.

 It is important to highlight the similarity between freedom from reintervention at 10 years between patients aged 60-69 and > 70; 94.1 and 93.3 respectively.^6^ It has been demonstrated that hospital mortality with mechanical prosthesis is superior to bioprosthesis.^3^ Long-term is also favoured by the latter.^12^

 Given the good long-term freedom from SVD, and considering the with the advent of new interventional devices we can treat SVD in patients unsuitable for surgery with good results,^13^ should we now prefer a bioprosthesis instead of a mechanical valve for patients between 65 and 70 years in the mitral position?

 Despite our interest in the study of long-term durability of biological prostheses, several limitations are present. First, the definition of SVD does not follow the cannon of the Akins guidelines and was only determined by reoperation or death due to the lack of echocardiographic data. Another limitation is the retrospective nature of this single-centre study.

## Conclusion

 Our 10-year freedom from SVD of 93.1% matches and even surpasses those previously reported.

 Prosthetic valve selection should not limit quality of life. Age at intervention is a determinant in prosthesis durability; as found in our results, patients < 60 years were more prone to SVD (*P* < 0.001). Although SVD is an important concern, freedom from anticoagulation remains an advantage over mechanical prostheses in older patient gropus.

 The Epic porcine xenograft in mitral position has demonstrated excellent durability and long-term outcomes; representing an excellent option for patients requiring MVR.

## Competing Interests

 None declared.

## Ethical Approval

 This retrospective analysis received institutional-review-board-permission number 2021/521. Approved 21^st^ of July 2022.
